# Closing the Water Balance with a Precision Small-Scale Field Lysimeter

**DOI:** 10.3390/s24072039

**Published:** 2024-03-22

**Authors:** Brad F. Lyles, Brad D. Sion, David Page, Jackson B. Crews, Eric V. McDonald, Mark B. Hausner

**Affiliations:** 1 Division of Hydrologic Sciences, Desert Research Institute, Reno, NV 89512, USA; 2 Division of Earth and Ecosystem Sciences, Desert Research Institute, Reno, NV 89512, USA; 3 Department of Earth, Marine, and Environmental Sciences, University of North Carolina—Chapel Hill, Chapel Hill, NC 27599, USA

**Keywords:** lysimeter, soil, vadose zone, evaporation, water balance

## Abstract

We developed a set of two precision, small-scale, water balance lysimeters to provide accurate measurements of bare soil evaporation. Each lysimeter comprises a soil tank, a balance assembly with load cell, a wicking drainage system, and a stilling well to measure drained water. Fiberglass wicks installed at the bottom of the soil tanks provide −60 cm of tension to the base of the soil column, and soil water drainage is quantified to close the water balance within the lysimeter. The calibrated lysimeters return mass changes with uncertainties ranging from 3 to 8 g, corresponding to uncertainties of 0.02–0.05 mm of water. Installed at a semi-arid site in northern Nevada, the two lysimeters are filled with uniform construction sand and silt loam. Over a six-month pilot observation period, bare soil evaporation rates of 0.19 and 0.40 mm/day were measured for the construction sand and silt loam, respectively, which is consistent with meteorological data and models of potential evapotranspiration at the site. The design of the lysimeter can be adapted to specific research goals or site restrictions, and these instruments can contribute significantly to our ability to close the soil water balance.

## 1. Introduction

Bare soil evaporative flux is a complicated physical process that has a profound effect on the soil water balance in the unsaturated zone. This is especially true in arid settings where soils are largely unvegetated. Evaporative flux from wet bare soil depends on meteorological conditions, while evaporation from dry surfaces is controlled by soil water resistance due to vertical vapor pressure gradients across a surface boundary [1], multiphase water movement [2], and soil texture [3], among others. Despite recent advancements in our understanding of the physics involved with bare soil evaporation, the process remains poorly understood. This is particularly true in semi-arid and arid environments [4] where limited moisture availability commonly prevents accurate quantification to help close the soil water balance. Soil evaporation models are especially unreliable at the dry end of the soil–water retention curve, where impediments to flow limit evaporation [5,6,7]. Thus, the simulation of soil evaporation and closure of the water balance in semi-arid and arid settings represents an ongoing challenge. A direct precision measurement of evaporation would substantially advance our ability to observe the entire soil water balance.

There are several techniques that are commonly used to measure evaporative fluxes. Eddy covariance (EC) techniques are popular because they provide direct meteorological observations of wind speed, relative humidity, and radiant fluxes that can be used to calculate bare soil evaporation; however, this approach is not appropriate for many applications due to the inherent issues that arise as a result of the spatial averaging area (extending several kilometers up fetch from the EC station). Water balance weighing lysimeters have been used for many years to measure evaporation, evapotranspiration [8], water stress in plants [9], crop-specific water use [10,11], and groundwater recharge [12], and they are commonly used to calibrate EC stations [13]. Weighing lysimeters therefore present a viable, high-precision alternative solution to compute bare soil evaporation. Fisher [14] reported on the design and construction of an electronic weighing lysimeter used to measure evapotranspiration and crop coefficients in agricultural settings that yielded good results, and recent literature includes reports of low-cost suspended lysimeters to measure water uptake by shallow rooting crops [15] and modular lysimeters for use with deeper-rooted crops [16]. However, no commercially available small-scale weighing water balance lysimeters can measure evaporative fluxes at the same precision and temporal resolution at which we measure other water balance components.

We document the development of a small-scale precision water balance lysimeter to improve the precision of evaporative flux measurements at a research site in northern Nevada. This paper details the fabrication and installation of two lysimeters at the site, their mechanical operation, and the sensitivity and reliability of the evaporation measurements. We provide preliminary field data from the two different research plots to demonstrate the capabilities and potential applications for future research.

## 2. Materials and Methods

### 2.1. Site and Soil Characteristics

**Figure 1:**
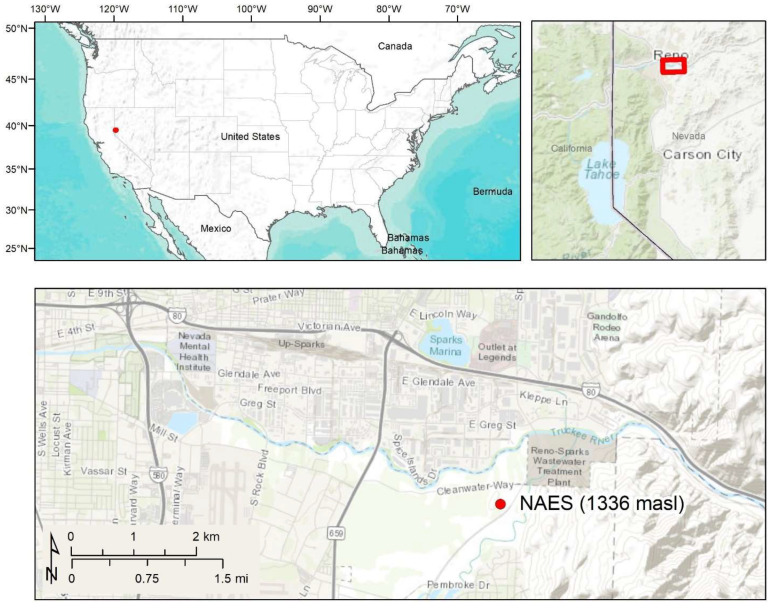
Location of the Nevada Agricultural Experiment Station study area.

The Nevada Agricultural Experiment Station (NAES) in northern Nevada (Figure 1) is an active agricultural site used by researchers across the University of Nevada community. Two lysimeters were deployed in a test bed used for the development and testing of remote sensing techniques to address applied research questions within our research group. The site is located within an agricultural field on a floodplain adjacent to the Truckee River. The local soil map units at the site (Voltaire silty clay and loamy sand) consist of deep, poorly drained soils classified as Fluvaquentic Endoaquolls. The typical Voltaire pedon contains a 25 to 50 cm thick mollic epipedon and redoximorphic concentrations in parent material horizons indicative of shallow water table conditions associated with a low-lying landscape position [17]. The site is on the boundary between the dry, high desert climate of the western Great Basin and the Mediterranean climate of the Sierra Nevada mountain range. Average annual precipitation is 190 mm, with two thirds of that falling between November and April. Annual snowfall is approximately 600 mm, mainly between November and March. Average daily temperatures range from 1 to 22 °C, with July highs averaging 33 °C and December lows averaging −6 °C [18].

**Figure 2:**
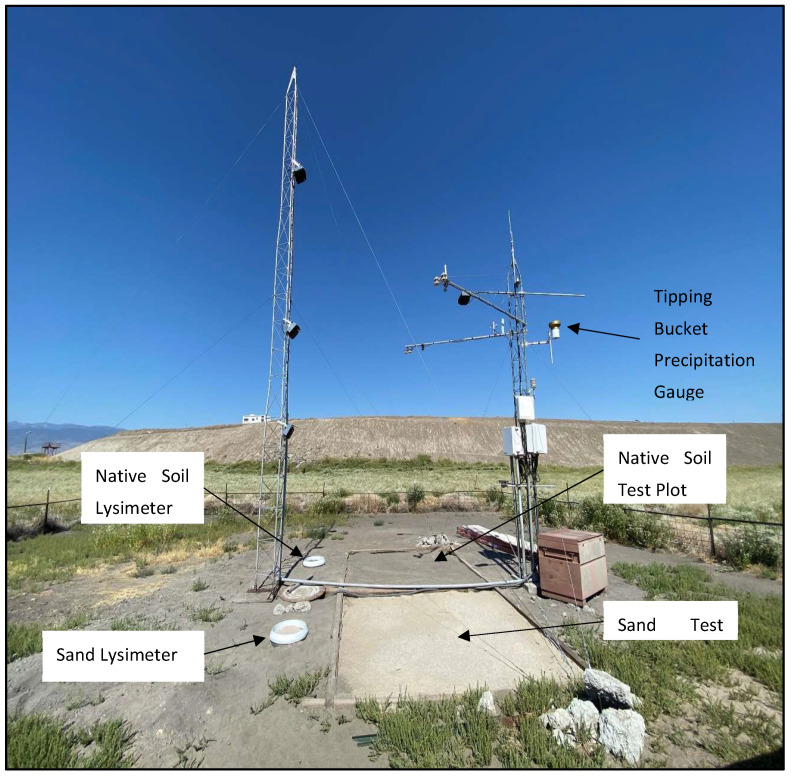
Photo of study site looking west; sand lysimeter (left foreground), sand test plot (right foreground), soil lysimeter (left mid-ground), soil test plot (right mid-ground) and meteorologic sensor tower (right).

The NAES site contains two soil test plots: one constructed from in situ soil material and a second from construction sand that respectively serve as “native” and “control” plots for research and development activities. The two 9 m^2^ (3 m length, 3 m width) soil test plots were instrumented to monitor near surface fluxes of heat and water at the site. Soil test plots were excavated to a total depths of 0.75 m, and sensors were positioned at various levels during construction while the pits were backfilled with soil material. The lysimeters constructed during this study were installed within 0.5 m of each test plot, at the same surface elevation and bare of vegetation, to match the conditions in the adjacent test plots (Figure 2). One test plot was filled with medium (predominant size range 0.3–0.8 mm) construction sand (Quikrete Companies, Atlanta GA, USA), and the other test plot was filled with disturbed native soil material. The associated lysimeters were filled with the same materials and are hereafter referred to as “sand lysimeter” and “soil lysimeter”, respectively. In this case, the experimental design dictated that the disturbed soil test plot was backfilled with native soil in a non-systematic manner; however, generally, the last soil out of the soil pit was the first soil back into the test plot.

We performed laboratory analyses of soil samples collected from the test plots after construction to characterize the physical and hydraulic properties of the materials. Particle size distribution analyses (PSDA) were performed on the fine-earth fraction (<2 mm) of each sample after pretreatments to remove soluble salts and organic material. PSDA was measured using ASTM C1070-01 [19] on a Malvern Mastersizer II. Results were binned to characterize the sand (2000–50 μm), silt (50–2 μm), and clay (<2 μm) particle size fractions (Table 1). Soil organic matter (SOM) contents were measured via loss-on-ignition following methods outlined by Storer [20]. Sand and soil hydraulic parameters were determined using KSAT, HYPROP, and WP4C devices (METER Group USA, Pullman, WA, USA) to characterize the soil water retention curves. The laboratory-derived characteristics for the material used during construction of the soil and sand lysimeters are summarized in Table 1.

The disturbed native soil used in the test plot is a silt loam with ~70% silt and ~17% sand compared to the nearly 100% sand content associated with the sand lysimeter. The native soil contains ~3 wt. % soil organic matter and has a bulk density of ~0.97 g/cm^3^ that likely resulted from excavation and backfilling without compaction. The sand material lacks organic matter and has a bulk density of ~1.5 g/cm^3^. The saturated hydraulic conductivity (Ks) for the native soil in the test plot varies by a factor of ~3 as a function of soil depth and is on the order of 100 cm/d, while the Ks of the sand is approximately one order of magnitude greater. The van Genuchten parameters presented in Table 1 reflect the greater water-holding capacity and broader dry-down curves associated with the native soil compared with the more uniform sand material. The two lysimeters were filled with native soil or sand and packed to match the material in the two test plots. Thus, these materials enable testing of the lysimeter design using two significantly different porous media.

### 2.2. Water Balance Lysimeter Components

**Figure 3:**
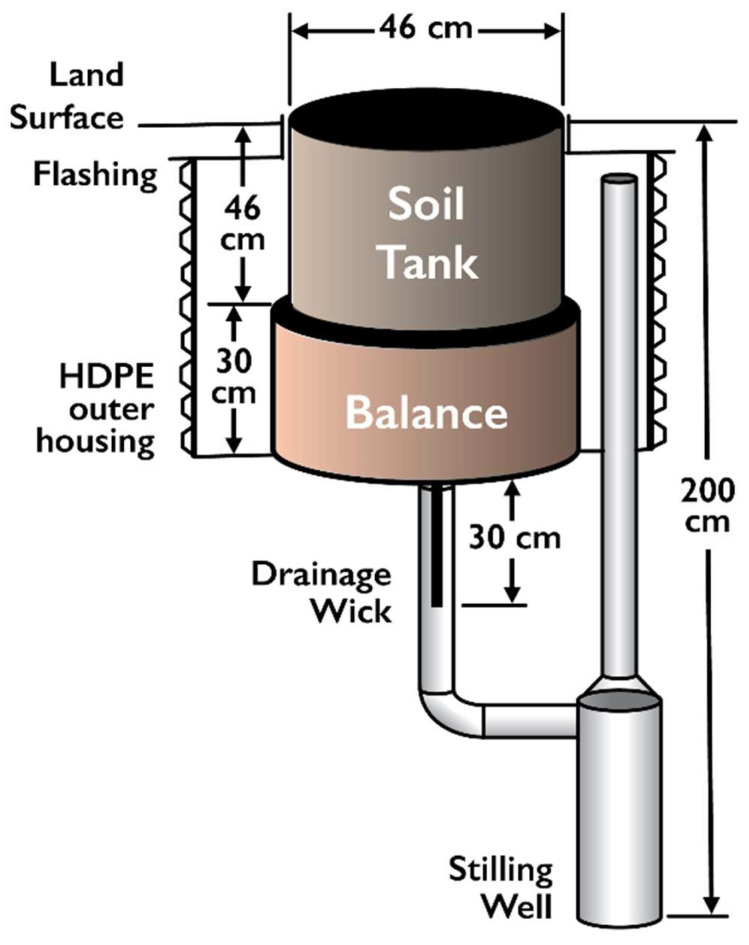
Precision weighing lysimeter major components are shown diagrammatically. The soil tank is filled with soil material and is positioned within 0.5 m of the test plot. The balance sits beneath the soil tank and is instrumented below with a drainage wick that is connected to a stilling well.

The water balance lysimeter design consists of three main components (Figure 3): (1) the balance mechanism with electronic load cell and counterweight, (2) a soil tank with soil moisture sensors and drainage wick, and (3) the drainage measurement system. The lysimeter is housed inside a vertically oriented, corrugated high-density polyethylene (HDPE) pipe.

**Figure 4:**
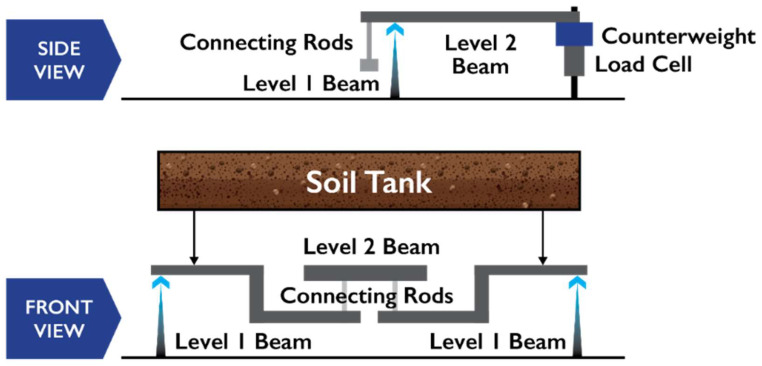
Balance mechanism diagram (the front view shows the soil tank mass is supported by two level 1 beams, the downward force is transferred via two connecting rods to the level two beam. The side view shows the downward force at the connecting rods producing an upward force at the load cell).

The steel beam balance mechanism produces a mechanical advantage to improve the resolution of the mass measurement, which is shown diagrammatically in Figure 4 (for lysimeter design details, see Appendix A). The lever-arms and fulcrums are configured to produce a 20 times mechanical advantage (i.e., a 20 g change in soil tank mass will result in a 1 g change in load cell mass) for this deployment. A ZA1-25 S-form load cell (Sentran, LLC, Ontario, CA, USA) is used to measure the change in force exerted on the level 2 beam. A counterweight is added at the load cell measurement point to neutralize the initial mass of the soil tank and to place the load cell in the appropriate range for the site conditions, allowing it to measure either losses or gains in mass. For example, if the soil moisture during lysimeter installation is in the mid-range, then counterweight mass should be added so that the load cell will read near the mid-range of the load cell.

The ZA1-25 electronic strain gauge load cell can measure ± 11.3 kg (25 pounds); the load cell is a “Wheatstone bridge” and its accuracy is limited by the datalogger measurement resolution. CR3000 dataloggers (Campbell Scientific Inc., Logan, UT, USA) were used to measure the output voltage from the load cell. The 5 V DC excitation voltage and 0.033 mV measurement resolution yield a theoretical load cell resolution of 0.14 g (0.0003 pounds).

The lysimeter size is a tradeoff between manufacturing cost, installation complexity and data resolution; this installation was designed to provide a short-term (months) comparison to the nearby test plots. The stainless-steel soil tank is 46 cm (18 inches) in diameter and 46 cm deep. A drainage wick is in contact with the soil at the bottom of the tank, and an integral funnel at the bottom focuses excess water to the wick. Drainage wicks have been used extensively in passive capillary samplers [21,22,23] and less frequently in passive soil water flux meters [24] and laboratory drainage lysimeters [25], but this is the first known use of a drainage wick in a continuously weighing field lysimeter. The wick provides soil water drainage at the bottom of the lysimeter without requiring the soil water content to reach field capacity. The wick is 80 cm long with 20 cm splayed out on the bottom of the soil tank and 60 cm hanging vertically below the lysimeter tank. The wick maintains an unsaturated tension of approximately −6 kPa at the bottom of each lysimeter tank. The hanging portion of the wick extends 30 cm below the base of the balance mechanism encased in a 12 mm inside-diameter stainless steel drainage tube that discharges to a 4-inch acrylonitrile butadiene styrene (ABS) pipe stilling well (10.2 cm inside diameter). Water level in the stilling well is monitored with a CS-451 submersible pressure transducer (Campbell Scientific, Inc.). The transducer, which has a measurement range of 0–20 kPa +/− 0.1%FS, can resolve ± 2 mm of water stage. The stilling well has a design capacity of 4 L at a stage of 50 cm. A Mini-Typhoon submersible sampling pump (Forestry Suppliers Inc., Jackson MS, USA) is triggered at a prescribed stage to remove water from the stilling well and discharge it to the ground below the test plot. The stilling well water volume versus stage calibration was performed by adding known incremental volumes of water to the stilling well after the transducer and submersible pump were installed.

The disturbed native soil lysimeter is equipped with two CS-655 soil moisture sensors (Campbell Scientific Inc.); the upper sensor is located 7.6 cm below the surface—the same depth as sensors inside the adjacent 3 by 3 m soil experiment plot. The lower sensor is located 2 cm above the drainage wick at 45.7 cm below the surface. Because the sand drained much faster than the soil, the sand lysimeter only has one CS-616 moisture sensor (Campbell Scientific Inc.) located 2 cm above the drainage wick.

### 2.3. Calibration and Uncertainty

Two whole-system field calibrations were performed on each lysimeter to account for manufacturing variations, load cell differences, and temperature dependencies. Calibrations were performed in July 2020, May 2021, and December–January 2023 to encompass both warm and cold temperatures. In each calibration process, calibration standards of known mass were placed on the soil surface, and the corresponding voltage changes were recorded every ten seconds by the datalogger. Calibration standards were added stepwise and were removed in the reverse order. To account for any systematic hysteresis, separate analyses were conducted for the addition (“wetting”) and subtraction (“drying”) of weight. After each weight was added, voltage readings were allowed to stabilize, and the average of the final six readings (final one-minute) was recorded. Trends in load cell voltage were assessed immediately before and after calibration, and any consistent trends (i.e., the same magnitude and direction before and after calibration) were removed from the datalogger voltages. The known masses were regressed against the average datalogger voltage to solve for the multiplier used in the datalogger program at the calibration temperature as well as the 95% confidence interval surrounding this multiplier. If the 95% confidence intervals for the wetting and drying analyses overlap, hysteresis is deemed negligible, and a single multiplier can be used. Calibration temperature was recorded by a thermocouple attached to the load cell itself.

The datalogger program was written so the lysimeter can be zeroed to initiate a new experiment. The initial mass is set as the tare mass, and the tare mass is removed from subsequent measurements, effectively zeroing the scale. Taring the lysimeter means that the regression intercept is not required. We compared the warm (May, July) and cold (December, January) calibrations to one another to determine whether a temperature correction was needed. If the regression between voltage and lysimeter mass showed a significantly different slope (i.e., if the 95% confidence intervals of the voltage-mass regressions did not overlap), we determined a temperature-dependent calibration in the form of

(1) $$\Delta M=\left(c_{T}T+c\right)V+M_{t},$$

in which ΔM is the cumulative change in mass since the lysimeter was tared, $c_{T}$ is a temperature correction factor, T is the load cell temperature as read by the thermocouple, c is the baseline multiplier, V is the load cell voltage read at the datalogger, and $M_{t}$ is the tare mass of the lysimeter. If the warm and cold slopes were not significantly different, we determined a static calibration in the form of

(2) $$\Delta M=cV+M_{t},$$

The root mean squared error (RMSE) of the calibrated measurements was computed from the calibration results for each lysimeter.

The ZA1-25 S-form load cells can resolve changes in weight as small as 0.14 g (limited by the datalogger measurement resolution) under ideal operating conditions. With the 20 times mechanical advantage, this resolution corresponds to a theoretical resolution of ±2.8 g of mass in the lysimeters. However, the field deployment and calibration add to this theoretical minimum. Converting the mass uncertainty to volume will add the temperature dependence of water density (±0.35%, assuming a temperature range of 0–40 °C). Converting that volume to a one-dimensional flux adds the uncertainty in area of the lysimeter (±4.5%, assuming an uncertainty of 0.5 cm in the nominal radius of the steel enclosure). We determined the overall uncertainty in the measurements of water depth by propagating all these individual uncertainties.

Wind can add uncertainty to the load cell observations, particularly when it is blowing parallel to the orientation of the beams that produce the mechanical advantage [26]. In this lysimeter, the two-level beam system (Figure 4) is designed to minimize the effect of a shear force exerted on the lysimeter surface—the shear force increases the load on one level-1 beam while decreasing it on the other. The net result is that the force transferred to the level-2 beam, which is attached to the load cell, remains undisturbed. To further mitigate this risk, the datalogger can be configured to use a running average of high-frequency mass observations rather than lower frequency sampling; this running average can minimize the effect of wind-driven oscillations in the load cell observations [26].

### 2.4. Water Balance Calculations

Evaporation was computed from each lysimeter by performing a mass conservation water balance calculation. We assume that all changes in mass are due to water entering or leaving the lysimeter and apply a sign convention that denotes mass entering the lysimeter as positive and mass leaving the lysimeter as negative. The mass balance takes the following form:

(3) $$\Delta M_{l}=\Delta M_{p}+\Delta M_{d}+∆M_{e},$$

where $\Delta M_{l}$ is the change in lysimeter mass, $\Delta M_{p}$ is the change in mass due to precipitation, $\Delta M_{d}$ is the change in mass due to lysimeter drainage, and $\Delta M_{e}$ is the net change in mass due to evaporation and condensation.

Precipitation was measured from a tipping bucket precipitation gauge installed on site, aggregating total precipitation in 15-min increments. The observed depth of precipitation is converted to units of mass using Equation (4):

(4) $$\Delta M_{p}=PA_{l}\rho ,$$

where $\Delta M_{p}$ is the lysimeter mass gain from precipitation, P is the depth of precipitation measured at the tipping bucket, $A_{l}$ is the surface area of the lysimeter, and ρ is the density of water.

Cumulative drainage $\Delta M_{d}$ was computed from the water level and cross-sectional area of the stilling well using Equation (5):

(5) $$\Delta M_{d}=A_{sw}(-\Delta h)\rho ,$$

where $\Delta M_{d}$ is the cumulative drainage from the lysimeter, $A_{sw}$ is the cross-sectional area of the vertical ABS stilling well, and Δh is the change in water level inside the stilling well. The negative sign in Equation (5) denotes that an increase in the stilling well stage h indicates drainage (i.e., a negative change in mass) from the lysimeter.

The lysimeter calibration, which includes the balance mechanism, mechanical advantage, load cell characteristics, and initial tare settings, translates the force measured by the load cell into a lysimeter mass $M_{l}$. The change in lysimeter mass $\Delta M_{l}$ is then calculated as the difference between successive readings of the lysimeter mass. With the individual components known, the water balance (3) is converted to one-dimensional water flux as

(6) $$E_{net}=\frac{\Delta M_{l}-\Delta M_{p}-\Delta M_{d}}{\Delta t\rho A_{l}},$$

where $E_{net}$ is the net evaporation rate (i.e., evaporation minus condensation) and Δt is the time over which all the components change in mass.

### 2.5. Field Deployment

**Figure 5:**
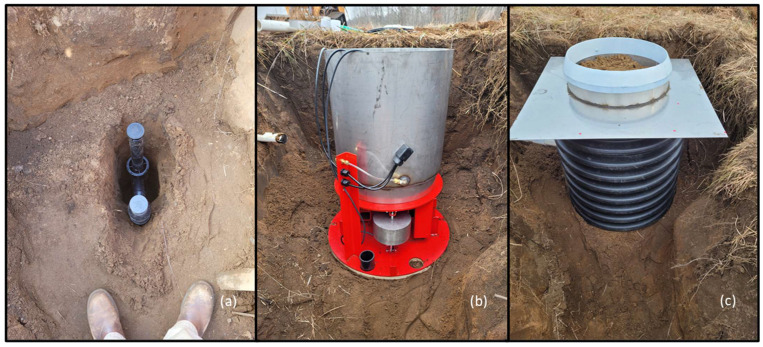
(**a**) Stilling well installed ~1.5 m below lysimeter balance, (**b**) installed lysimeter, balance (red) with counterweight (silver) (note 2” ABS stilling well riser pipe), stainless steel soil tank, with two soil sensor cables (black) on the side of the tank (temporarily looped over the top of the tank until the HDPE is installed) and wick calibration line (clear tubing), and (**c**) complete lysimeter with HDPE outer shell installed with surface flashing (silver) and lysimeter ring flashing (white) installed.

Two lysimeters were deployed adjacent to the test plots that were described previously. Two shallow soil pits were hand excavated approximately 90 cm diameter by 75 cm deep to house the lysimeter balance and soil tank. A deeper hole was dug near the center of the soil pits for placement of the stilling well and soil drainage pipes (Figure 5a). Once the drainage plumbing was installed, the pit floor was compacted and leveled. Each lysimeter was installed in the soil pit, ensuring the balance was centered on the drainage pipe (Figure 5b). The lysimeter balance assembly and soil tank were housed inside a 76 cm (30 inch) diameter HDPE pipe. The soil tank and drainage wick were installed onto each balance. Stainless steel flashing, installed to reduce the influence of wind on the lysimeter surface, rests on top of the HDPE with a 1 cm air gap between the flashing and the soil tank. Soil was backfilled and compacted around the outside of the HDPE. The metal flashing was covered with soil, and a circular ring flashing was installed to keep debris from entering the lysimeter enclosure (Figure 5c). The ring flashing has approximately 1 cm freeboard to the soil surface to the bottom of the flashing. Periodic maintenance is needed to ensure soil and vegetation do not reach the bottom of the ring flashing and that vegetation does not accumulate inside the lysimeter tank (Figure 2).

## 3. Results

### 3.1. Field Calibration and Uncertainty

**Figure 6:**
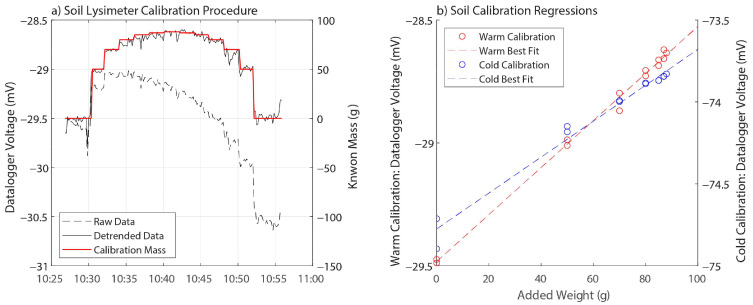
(**a**) Example of soil lysimeter calibration process with raw (dashed black line) and detrended (solid black line) load cell potential readings on the left y-axis and the calibration known mass values (solid red line) on the right y-axis. (**b**) Linear regression results of load cell potential (y-axis) versus mass added to the lysimeter using calibration standards (x-axis) for warm and cold calibrations.

**Figure 7:**
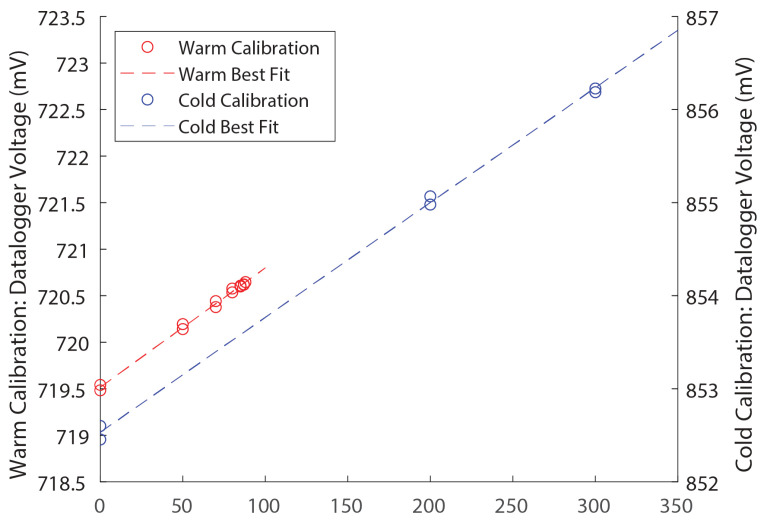
Sand lysimeter regressions for warm (red) and cold (blue) calibrations. Note that the slopes of the two regression lines are nearly identical as opposed to the warm and cold soil calibration regressions shown in Figure 6b.

Datalogger voltages measured for different known calibration masses are shown in Figure 6 and Figure 7. Calibration data are shown below in Table 2.

In both the sand and the soil lysimeters under both warm and cold conditions (Figure 6a and Figure 7), the regression slopes for the added weight (wetting) versus the weight removal (drying) were slightly different, but the 95% confidence intervals of the two slopes overlap (Table 2), indicating that hysteresis was not significant to the calibration. The calibration slope for each row of Table 2 was therefore determined as a single number to be applied to both increasing and decreasing mass. Hysteresis does not affect the calibration to the degree that wetting and drying periods require different calibration constants.

The soil lysimeter (Figure 6) was shown to have a temperature-sensitive calibration—note that the warm and cold regression lines in Figure 6b have two different slopes. Those slopes, along with the 95% confidence intervals around each slope, are shown in the second and fourth lines of Table 2. The warm slope (104.06 g/mV) falls outside of the 95% confidence interval of the cold slope (80.68–98.01 g/mV), and the cold slope (89.34 g/mV) falls outside of the 95% confidence interval for the warm slope (99.16–108.96 g/mV). This indicates that a temperature correction as described in Section 2.3 above is necessary for the soil lysimeter. The soil lysimeter calibration therefore takes the form of Equation (1) above. The sand lysimeter does not require a temperature correction (i.e., the 95% confidence intervals of the warm and cold calibrations overlap), and the sand lysimeter calibration takes the form of Equation (2). Final calibration parameters for both lysimeters are shown in Table 3.

The uncertainty of mass changes measured by the calibrated lysimeters (as quantified by the RMSE) is ±3.6 g in the sand lysimeter and ±7.8 g in the soil lysimeter (Table 3). We added that uncertainty to the theoretical uncertainty of the load cell (±2.8 g, as noted in the Methods section) and converted that to an uncertainty in vertical depth of water (accounting for temperature-dependent water density and uncertainty in the surface area of the lysimeters. The propagation of all these individual uncertainties results in an overall uncertainty that corresponds to ±0.02 mm of water in the sand lysimeter and ±0.05 mm of water in the soil lysimeter.

### 3.2. Field Observations

**Figure 8:**
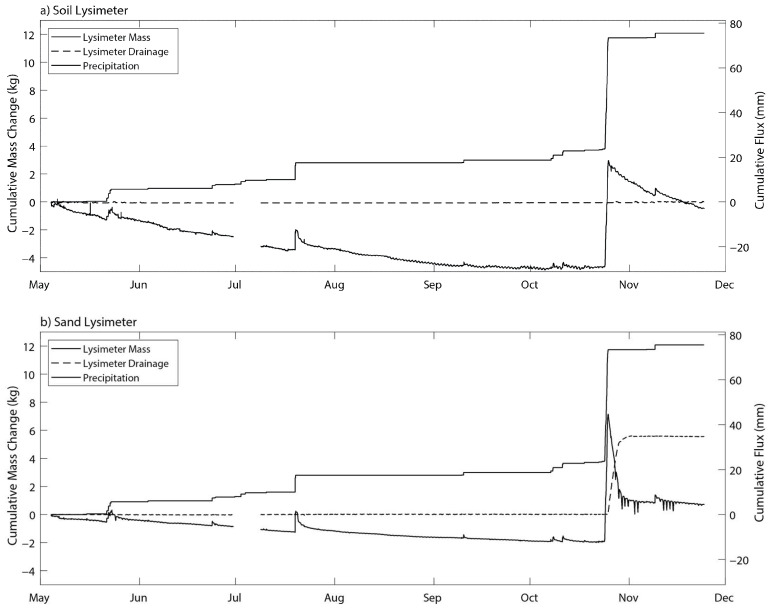
Time-series of changing mass from the (**a**) soil and (**b**) sand lysimeters, cumulative drainage from each lysimeter and cumulative precipitation; 5 May to 24 November 2021.

The deployed lysimeters recorded changes in lysimeter mass and drainage in the test plots every 15 min from 4 May 2021 to 24 November 2021 (Figure 8). Changes in mass recorded by the lysimeters were compared to an existing TE-525MM precipitation gauge (Texas Instruments) already located at the site.

The soil moisture sensors near the drainage wick are used to determine that the wicking drainage system is working properly (for example, soil moisture greater than the soil field capacity would indicate water is not draining properly). The shallow soil moisture measured in the soil lysimeter compared well to the shallow soil moisture measured in the study plot; details are shown in Appendix B. Drainage was observed from the sand lysimeter following the 50 mm precipitation event on 24 October 2021 with a concurrent decrease in the lysimeter mass. In the soil lysimeter, however, no drainage was observed between May and November; the precipitation that fell during the study period was either lost to evaporation or remained stored in the lysimeter soil moisture.

## 4. Discussion

In this section, we examine more closely individual phenomena observed by the lysimeters. We consider specifically evaporation, response to precipitation events, and diurnal behavior during a dry period.

### 4.1. Evaporation

**Figure 9:**
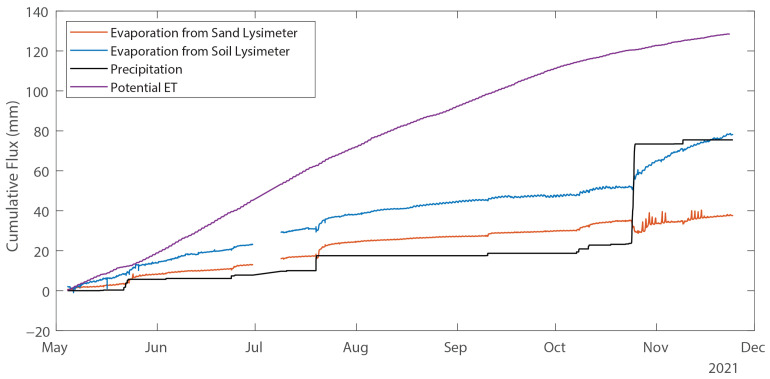
Time-series of cumulative precipitation, PET and evaporation from each lysimeter (all expressed as mm of water).

The cumulative evaporation, precipitation, and potential evapotranspiration (PET) for the period of record (4 May to 24 November 2021) are shown in Figure 9. The PET was computed with the computer program RefET for FAO56 [27] using an onsite meteorological station and a crop coefficient Kc = 0.15 for bare soil; the Kc is a literature-reported value, and the PET is only shown for reference to the actual evaporation measured from the lysimeters. The soil lysimeter evaporated 76 mm of water compared to 35 mm from the sand lysimeter; both are well below the 128 mm potential evapotranspiration over the full period. Over the same time, there was 75.5 mm of precipitation observed, most of it coming in the single 24–25 October 2021 event described above. Prior to that event, cumulative evaporation from the sand lysimeter was slightly greater than cumulative precipitation for the period of record, suggesting that little water is stored in the sand. Conversely, no drainage had been observed from the soil lysimeter, and all evaporation had been drawn from either precipitation or soil water storage. Prior to the single storm event, cumulative precipitation was approximately 20 mm and cumulative evaporation from the soil lysimeter was approximately 55 mm, indicating that the soil lysimeter retains water and evaporation occurs from storage in addition to precipitation. Even if precipitation was underestimated by 40% [28], the soil lysimeter would have contributed half of the evaporation from storage.

### 4.2. Precipitation Events

**Figure 10:**
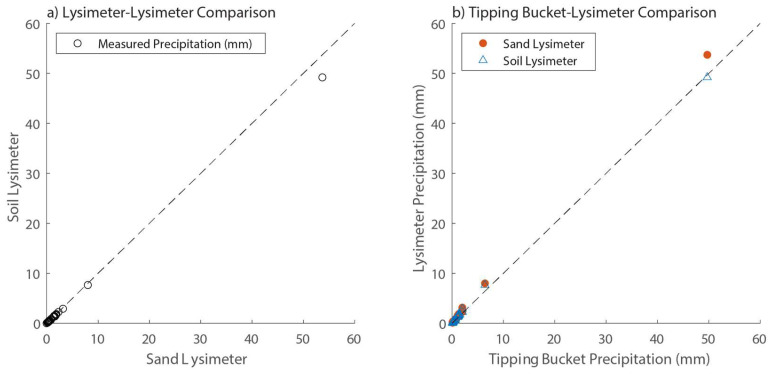
Scatter plots of observed precipitation. (**a**) Precipitation observed at the sand lysimeter (x-axis) and the soil lysimeter (y-axis). (**b**) Precipitation observed at the TE-525 tipping bucket (x-axis) and at the lysimeters (y-axis). In each plot, the 1:1 line is indicated by the dashed black line.

The response of the lysimeters to individual precipitation events is shown in Figure 10. The on-site tipping bucket recorded 75.5 mm of precipitation during 27 different precipitation events over the course of this study with individual events ranging from 0.1 mm over a 15-min period to 50.1 mm over 29-h duration event. Just one event recorded more than 8 mm of precipitation. The lysimeters responded consistently to all but the largest precipitation event with the sand lysimeter measuring approximately 3.5% more precipitation than the soil lysimeter (Figure 10a). During the largest event, however, the sand lysimeter registered 9.2% more precipitation than the soil lysimeter, and the data point for this event is the only one that falls significantly off the 1:1 line (Figure 10a).

The largest event was also an outlier when the lysimeters were compared to the on-site tipping bucket (Figure 10b). During the 26 smaller events, the sand and soil lysimeters registered 26.8% and 22.5% more precipitation than the tipping bucket, respectively, and the lysimeter data points consequently fall above the 1:1 line shown in Figure 10b. This is not surprising given that tipping bucket rain gauges are known to under-report precipitation by 5–40%, varying by season and setting [28,29,30]. In the large 24–25 October event, however, the sand lysimeter measured 8% more precipitation than the tipping bucket (within the range seen during earlier events), whereas the soil lysimeter measured 1% less precipitation than the on-site gauge. Because the soil lysimeter behaved differently when compared to both the sand lysimeter and the tipping bucket, we hypothesize that the soil lysimeter was most likely over-topped by the intensity and duration of the event.

**Figure 11:**
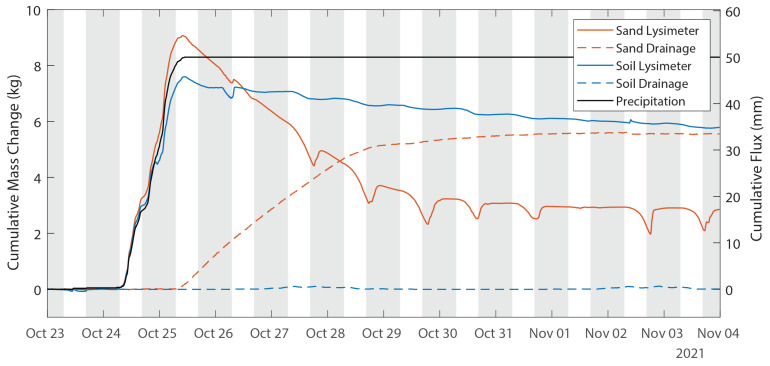
Time-series of lysimeter response to a precipitation event occurring 24–25 October. Shaded areas on the plot indicate nighttime hours.

The lysimeter response to this precipitation event is shown in more detail in Figure 11. The on-site tipping bucket rain gauge reported 50.0 mm, the sand lysimeter reported 54.2 mm and the soil lysimeter reported 49.3 mm. The accuracy of the tipping bucket is reported by the manufacturer as ±1% for precipitation rates up to 50 mm/h, but Hoffman et al. [29] recently found that a tipping bucket precipitation gauge 1 m above the ground underestimated cumulative precipitation by more than 12% when compared to precision lysimeters and noted that the error increases with the height of the tipping bucket. At this site, the tipping bucket was installed 3 m above the ground. Because the sand drains much faster than the soil, it was able to infiltrate the full volume of precipitation, whereas the soil lysimeter overflowed and allowed some water to leave the system unmeasured. In the soil lysimeter, the upper soil moisture sensor showed a sharp increase in soil moisture through the storm, peaking approximately six hours before the peak in mass, while the lower soil moisture sensor did not show a response to the storm event until 26 October, two days after the event began. This is consistent with a soil that has reached its infiltration capacity and cannot take in any more water. No drainage occurred from the soil lysimeter; however, nearly all the sand lysimeter decrease in weight can be attributed to drainage. Nightly increases in mass are observed in both lysimeters but are more prominent in the sand lysimeter than in the soil lysimeter (Figure 11).

**Figure 12:**
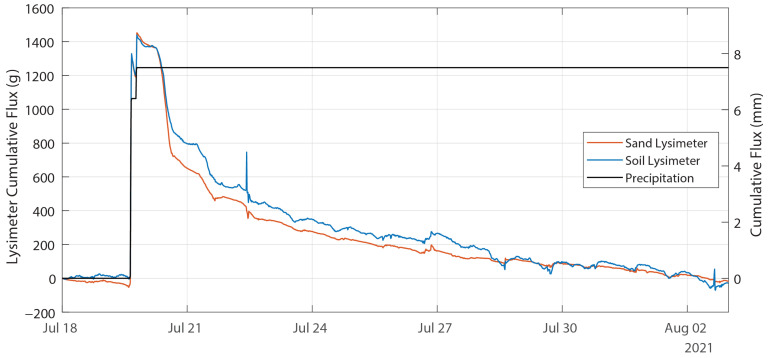
Time-series of lysimeter response to a smaller precipitation event occurring 19 July. Cumulative lysimeter fluxes are shown in mass on the left axis and mm of water on the right axis, and the cumulative precipitation is shown in mm of water on the right axis.

That large precipitation event can be contrasted with the smaller event observed on 19 July. Figure 12 shows the cumulative change in water stored in the lysimeters and the cumulative precipitation measured by the tipping bucket rain gauge during this event. The two lysimeters both measured 8.8 mm of precipitation, which is 17% more than the tipping bucket measurement of 7.5 mm. In contrast to the larger event described above, there was neither drainage nor apparent overtopping or runoff from either lysimeter. The water introduced during the event was lost to evaporation within two weeks, as indicated by the cumulative change in lysimeter storage shown in Figure 12 returning to zero on 2 August. The observations from the lysimeters installed on this site make it possible to partition the observed precipitation into runoff, evaporation, storage, and infiltration into the deeper soil profile.

### 4.3. Diurnal Behavior during Dry Periods

**Figure 13:**
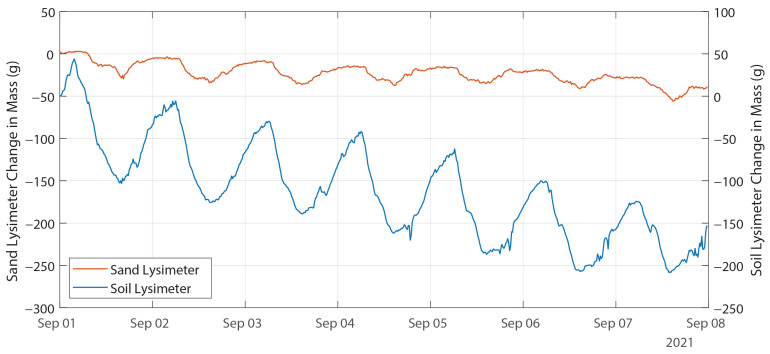
Time-series plot of lysimeter response to no precipitation, 1–8 September 2021.

An example of lysimeter behavior during a dry period is shown in Figure 13. No lysimeter drainage occurred during the 7-day dry period. Both lysimeters showed decreasing mass, with the sand lysimeter reporting −3.6 g/day and the soil lysimeter reporting −17.6 g/day (0.022 mm/day and 0.107 mm/day, respectively)—evaporation from the soil is an order of magnitude higher than evaporation from the sand. Both lysimeters decrease mass during the daylight hours (7:00 to 16:15) and increase in mass during the late afternoon and night (16:30 to 6:45). This diurnal pattern is consistent with dewfall observed by other researchers [31,32].

**Figure 14:**
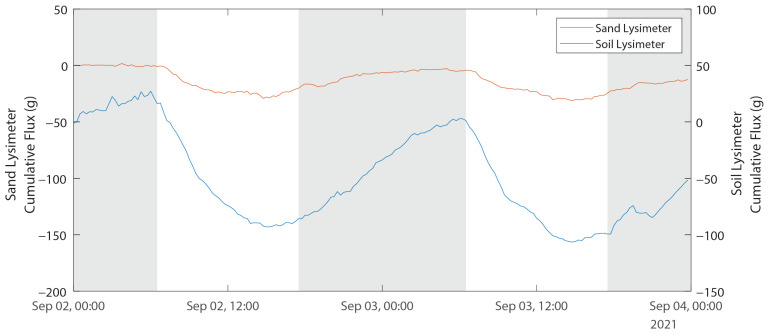
Time-series plot of lysimeter response to no precipitation, 2–3 September 2021. The shaded areas in the plot indicate night.

Dewfall is more obvious on shorter duration (Figure 14), and with day/night as bars on the same graph. Linear regression over that 48-h period shows time-averaged mass loss rates of 4.3 g/day (0.026 mm/d) and 22.6 g/d (0.14 mm/d) in the sand and soil lysimeters, respectively. Despite the very low average evaporation rates from both lysimeters, we were able to observe an order-of-magnitude difference between the two lysimeters in both the average evaporation rates and the daily amplitude of mass changes. These differences would not have been detected with other, less sensitive instruments.

## 5. Conclusions

The two lysimeters described here allow observations of precipitation, evaporation, and drainage at resolutions comparable to more expensive commercially available instruments. Although they are just a few meters apart at the same elevation on the same site and are subjected to the same environmental conditions, the large differences observed in both drainage and evaporation will lead to very different conditions in the two porous media. These conditions have implications for not only the soil water balance but also for agriculture and soil ecology. The lysimeters described here, which are simultaneously weighing and draining lysimeters, accurately capture the distinctive behavior of two very different media. The use of this weighing lysimeter makes it possible to close the water balance for a soil profile at this site.

Ongoing measurements will be further evaluated as more data are collected, but the results from the precision small-scale weighing lysimeters are reasonable and are consistent with theoretical expectations and other on-site instruments. Future installations may incorporate design changes based on the needs of the project. In humid or coastal areas, for example, materials should be selected with rust or corrosion resistance in mind. Similarly, different soil types may require design modifications as well. A deeper soil tank could offer better estimates of evaporation from finer textured soils and allow for more soil moisture sensors to better understand the distribution of soil water storage. A simplified balance mechanism could reduce costs while maintaining accuracy, and insulating the balance mechanism could reduce temperature effects at the load cell.

## Figures and Tables

**Table 1:** Physical and hydraulic properties of the disturbed Voltaire silt loam and sand at the NAES study site for three depth intervals.

Sample Material	Depth cm	Sand %	Silt %	Clay %	Organic Matter g/g	Bulk Density g/cm^3^	*K_s_* m/d	*θ_s_* m^3^ m^−3^	*θ_r_* m^3^ m^−3^	α -	*n* -
Native soil	0–5	16.9	70.1	13.0	3.2	0.96	1.25	0.593	0.067	0.030	1.354
	5–10	18.7	69.9	11.4	3.1	0.95	2.68	0.620	0.062	0.008	1.585
	10–15	17.6	70.8	11.7	2.7	0.98	3.24	0.561	-	0.085	1.200
Sand	0–5	97.8	2.1	0.1	0.1	1.51	48.6	0.315	0.016	0.049	8.397
	5–10	100.0	0.0	0.0	0.1	1.42	45.3	0.292	0.017	0.040	8.775
	10–15	100.0	0.0	0.0	0.0	1.43	62.1	0.220	-	0.045	10.913

**Table 2:** Calibration results for varying conditions.

Lysimeter	Temperature °C	Phase	Slope g/mV	95% CI g/mV	Slope g/mV	95% CI g/mV
Sand	23.1	Wetting	79.74	75.80–83.68	77.72	74.59–80.84
		Drying	76.13	73.96–78.30		
Soil	19.0	Wetting	105.93	101.58–110.27	104.06	99.16–108.96
		Drying	102.08	91.79–112.37		
Sand	1.9	Wetting	79.61	76.35–82.87	80.97	78.03–83.90
		Drying	82.21	78.93–85.49		
Soil	3.7	Wetting	82.75	72.47–93.03	89.34	80.68–98.01
		Drying	100.35	91.41–109.29		

**Table 3:** Whole system calibration and uncertainty parameters.

Lysimeter	*c* (mV g^−1^)	*c_T_* (mV g^−1^ °C^−1^)	RMSE (g)
Sand	79.44	N/A	3.6
Soil	85.78	0.96	7.8

**Figure A1:**
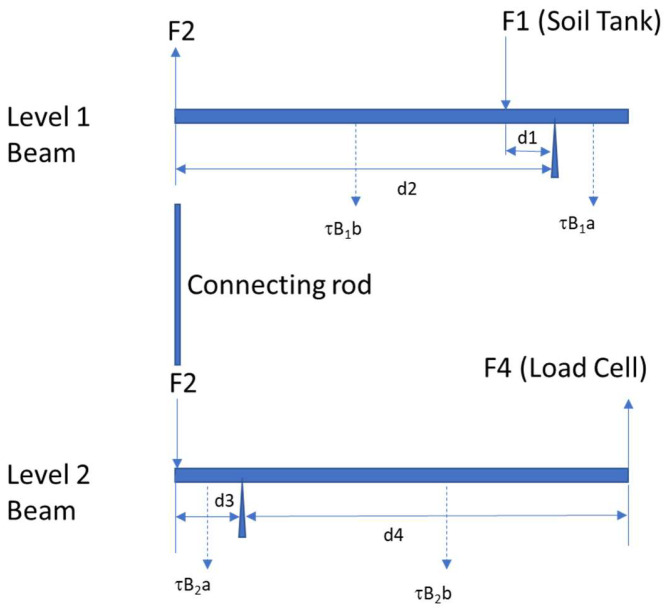
Beam lever-arm torque calculation diagram; soil tank load supported by the level-one beam lever-arm (*F*_1_), the load (*F*_2_) is transferred to the level-two beam lever-arm via connecting rods, and the resulting load (*F*_4_) is measured by the load cell.

**Figure A2:**
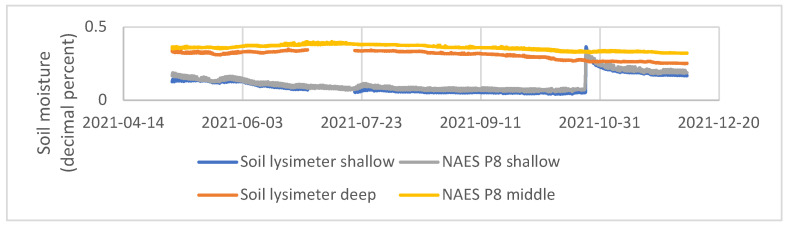
Time-series of soil volumetric moisture content in the lysimeter and the NAES adjacent soil test plot.

**Figure A3:**
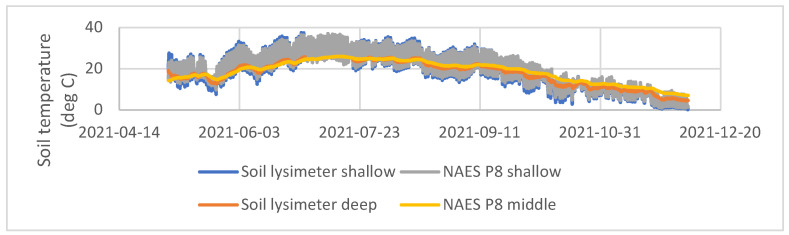
Time-series of soil temperature in the lysimeter and NAES adjacent soil test plot.

## Data Availability

We maintain a repository of design drawings and fabrication details for the lysimeter at https://github.com/lylesimeter. Data described in this article can be downloaded from DataDryad at https://doi.org/10.5061/dryad.15dv41p44.

## Appendix A. Water Balance Lysimeter Design

The lysimeter balance was designed based on the soil bulk density, soil porosity and soil water mass at field capacity; using engineering conservative estimates (1.8 g/cm^3^, 30% and 50% respectively), the design load of 194.1 kg was used. The lysimeter balance is designed to fit inside a 61 cm (24 inch) diameter corrugated HDPE outer housing; the outer housing keeps the surrounding soil from caving in on the lysimeter balance and soil tank.

Torque calculations determine the design mechanical advantage. The level-one beam torque is calculated according to Equation (A1):

(A1) $$F_{2}d_{2}={(F}_{1}d_{1}-\tau B_{1}a+\tau B_{1}b)$$

where *F*_2_ is the resulting force from the level-one beam, *d*_2_ is the distance the connecting rod to the fulcrum, *F*_1_ is the force from the soil tank load, *d*_1_ is the distance from the load to the fulcrum, *τB*_1_*a* is the torque produced by beam-one from the mass on the right side of the fulcrum and *τB*_1_*b* is the torque produced by beam-one from the mass on the left side of the fulcrum (the torque produced from the beam is small compared to the load, but it is included here for completeness).

The resulting mass at position *F*_2_ is transferred via connecting rods to beam-two, and the torque calculations were performed with Equation (A2).

(A2) $$F_{4}d_{4}=F_{2}d_{3}+\tau B_{2}a-\tau B_{2}b$$

where *F*_4_ is the final force at the load cell, *d*_4_ is the distance from the load cell to the fulcrum, *F*_2_ is the force transferred from the level-one beam, *d*_3_ is the distance from the connecting rods to the fulcrum, *τB*_2_*a* is the torque produced by beam-two from the mass on the left side of the fulcrum and *τB*_2_*b* is the torque produced by beam-two from the mass on the right side of the fulcrum. The beams are shown diagrammatically in Figure A1.

The soil tank mass is supported by two, level-one beam lever arms. Two connecting rods transfer the load to a single, level-two beam lever arm, which is connected to a load cell. The fulcrums are positioned relative to the length of the lever arms to produce the mechanical advantage. The values used in the balance design are listed in Table A1.

The connecting rod positions can be adjusted in the field to provide 20, 25, 29, 35, 42, or 50 times mechanical advantage to best suit the scientific requirements of the specific deployment (for example, if a larger soil tank is used, at higher soil bulk density or if used for snow water equivalent measurements).

**Table A1:** Torque calculations for beam lever arms and computed mechanical advantage.

**Beam 1**		
Design Load	194.1	kg
*F*_1_	1903.75	N
*F*_2_	−381.13	N
*d*_1_	0.025	m
*d*_2_	0.127	m
*t*_1_	48.36	Nm
*t*_2_	48.40	Nm
*τB*_1_ *a*	−0.002	Nm
*τB*_1_ *b*	0.050	Nm
**Beam 2**		
Design Load	194.1	Kg
*F*_1_	−381.13	N
*F*_2_	95.30	N
*d*_1_	0.076	m
*d*_2_	0.305	m
*t*_1_	−29.04	Nm
*t*_2_	−29.05	Nm
*τB*_1_ *a*	0.0004	Nm
*τB*_1_ *b*	−0.0061	Nm

## Appendix B. Comparison to Adjacent Field Plot

The shallow soil moisture compared well between the soil lysimeter and the study plot, but the lysimeter deep sensor showed lower volumetric moisture content than the test plot (Figure A2). The center of the NAES study plot is 2 m away from the lysimeter, and the P8 middle sensor is 41 cm deep. The shallow soil temperature compares well between the soil lysimeter and soil test plot, while the deep lysimeter temperature is cooler than the soil test plot (Figure A3).

The CS-655 soil sensor used in the sand lysimeter does not report soil temperature and the soil moisture has only changed slightly during the study period (ranging from 0.011 to 0.036); therefore, no comparison will be made with the adjacent NAES sand plot.
